# Implantable hemodynamic monitoring and management of left ventricular assist devices: Optimal or optional?

**DOI:** 10.1016/j.xjon.2021.09.026

**Published:** 2021-09-24

**Authors:** Brent C. Lampert, Jeffrey J. Teuteberg

**Affiliations:** a Division of Cardiovascular Medicine, The Ohio State University Wexner Medical Center, Columbus, Ohio; b Division of Cardiovascular Medicine, Stanford University Medical Center, Palo Alto, Calif

**Keywords:** mechanical circulatory support, left ventricular assist device, hemodynamics, implantable hemodynamic monitoring

**Figure:**
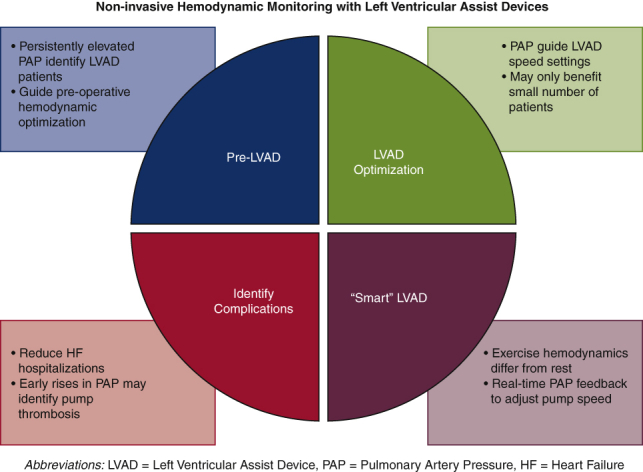
Potential uses of noninvasive hemodynamic monitoring with left ventricular assist devices.

**Central Message**

There is insufficient evidence for the routine use of implantable hemodynamic monitors in left ventricular assist devices, but they hold promise to improve pump performance and reduce adverse events.

See Commentaries on pages 24 and 26.

Continuous flow left ventricular assist devices (LVADs) improve quality of life and survival in patients with advanced heart failure.^1^, ^2^, ^3^ Despite these advances, LVADs are still limited by numerous complications, including frequent rehospitalizations, right ventricular (RV) failure, gastrointestinal bleeding, and hemocompatibility-related adverse events.^4^ Although continuous-flow LVADs are responsive to preload and afterload, their set speed, and thus their ability to properly unload the LV, is determined by clinicians. Further, loading conditions are influenced by medical therapies such as diuretics and vasodilators whose use and dosages are also under the discretion of the treating physician. Although there are general recommendations about pump management and medical therapy while on mechanical support, practices, therapeutic targets, and frequency of assessments may vary considerably between and even within centers.^5^ Despite these variations in practice, device and medical management can have a substantial influence on the development of major adverse events.^6^^,^^7^ Consequently, there has been increasing interest in developing strategies to optimize patients' functioning while on mechanical support in an effort to reduce adverse events.

The role of an LVAD is to continuously unload the LV and provide a more physiologic cardiac output. Increases in LVAD speed decrease end-diastolic volume and pressure and increase cardiac output and aortic pressures; thus, the choice of pump speed has a profound influence on the effectiveness of mechanical support.^8^ At low pump speeds, the LV has inadequate unloading and LV end diastolic pressure remains elevated, resulting in continued mitral regurgitation, the persistence of pulmonary hypertension, RV dysfunction, and dyspnea with exertion. Conversely, whereas high pump speeds reduce LV end diastolic pressures, they may be lowered to the point where the LV systolic pressure cannot overcome systemic pressures such that the aortic valve (AV) remains closed throughout the cardiac cycle, increasing risk for AV fusion, thrombus formation, and aortic insufficiency.^9^ Additionally, overdecompression of the LV can result in shift of the intraventricular septum and incite or worsen preexisting RV function, or trigger ventricular dysrhythmias via suction events.

The optimal LVAD pump speed maximizes LV unloading and cardiac output while not adversely influencing the position of the interventricular septum or leading to suction, ideally while maintaining some opening of the AV. The traditional method of optimizing pump speed entails an echocardiographic ramp study where pump speed is increased over a series of fixed increments while monitoring LV size, septal position, mitral regurgitation, and AV opening. Although these optimization goals are widely accepted, there are not generally agreed upon echocardiographic criteria to define adequate unloading. Careful echocardiographic assessments during ramp studies have found that the ability to influence some of these targets—such as change in LV end diastolic diameter—may differ based on pump type and negatively influence the utility of echocardiographic optimization.^10^^,^^11^

Given the limitations of echocardiographic optimization, there has been growing utilization of ramp studies based on invasive hemodynamic monitoring to select LVAD speed. Uriel and colleagues^12^ evaluated 35 patients with continuous-flow LVADs who underwent both hemodynamic and echocardiographic ramp studies. During the hemodynamic ramp study, Doppler blood pressure, central venous pressure, pulmonary artery pressure (PAP), pulmonary capillary wedge pressure, and cardiac output were monitored during progressive pump speed changes. At baseline, 43% of patients had optimal hemodynamic status characterized by a central venous pressure and pulmonary capillary wedge pressure that were both in the normal range at a pump speed previously determined by echocardiography. After the hemodynamic ramp, the proportion of patients who met these criteria for optimal hemodynamic status increased, but only modestly, to 56%.

The influence of invasive hemodynamic ramp testing on subsequent adverse events was assessed in 88 patients with continuous-flow LVADs of whom 44 were found to have optimal hemodynamic status at baseline.^13^ After the hemodynamic ramp study, 54 were categorized as having optimal hemodynamic status. Notably, 6 patients whose baseline hemodynamic status was optimal were categorized as nonoptimal after the ramp study. Compared with the nonoptimized patients, the optimized patients had a significantly higher freedom from readmission over the following year (44% vs 21% [*P* = .003]). Additionally, the overall readmission rates as well as readmissions related or not related to heart failure were significantly lower in the optimized group.

Given these findings, the strategy of invasive hemodynamic ramp testing was then tested in a randomized fashion against echocardiographic ramp testing in the RAMP-IT-UP trial of 41 HVAD (Medtronic, Minneapolis, Minn) patients.^14^ The hemodynamic ramp group had twice as many LVAD speed changes per patient, although the mean speed change per patient was <200 RPM. Perhaps more importantly, the hemodynamic group had twice as many changes in heart failure medications, particularly in beta-blockers and diuretics. Overall, the invasive hemodynamic group had numerically, but not statistically significant, lower rates of adverse events and higher event-free survival. There were no differences between groups in measures of quality of life or functional capacity. Despite the aforementioned increased number of speed and medication changes in the hemodynamic ramp group, 67% of patients had optimized hemodynamic parameters at baseline and this did not significantly increase after the hemodynamic ramp study. The optimal timing and strategy to address the one-third of patients whose hemodynamic status remained suboptimal before and after a hemodynamic ramp remains unclear and awaits the results of future investigations.

In light of the large percentage of patients with optimal hemodynamic status using echocardiographic assessment alone coupled with the inconvenience, cost, and risk, the invasive approach has not been widely adopted for routine optimization. However, for patients with recurrent readmissions, particularly for unexplained device alarms or heart failure symptoms, invasive hemodynamic ramp testing remains a very useful diagnostic tool to guide adjustments to pump speeds and medical therapy. Unfortunately, invasive hemodynamic ramp studies only provide a snapshot of the hemodynamic state of patients at a particular moment in time. With changes in loading conditions, patient position, and activity, hemodynamic status may vary considerably over time and hence a more longitudinal perspective on hemodynamic status may be of greater utility in the LVAD population.

In chronic heart failure populations, hemodynamic parameters have also been used to guide patient management, particularly when heart failure is in its advanced stages. Similar to the LVAD population, for whom periodic invasive hemodynamic assessments may be of diagnostic benefit, they are not routinely used for patient management. However, the development of implantable hemodynamic monitors (IHMs) have shifted the paradigm and allowed for the noninvasive, continuous, and remote monitoring of patients. CardioMEMS (Abbott, Abbott Park, Ill) is a wireless, IHM percutaneously implanted in a distal branch of the PA (Figure 1). Remote PAP-guided management of patients with heart failure using CardioMEMS reduces heart failure hospitalizations, improves cardiac filling pressures, and facilitates more aggressive titration of diuretics and neurohormonal antagonists.^15^ Given the potential benefits of hemodynamic optimization in patients with an LVAD, there has been growing interest in how this technology may be applied in mechanical circulatory support.^16^^,^^17^

**Figure 1:**
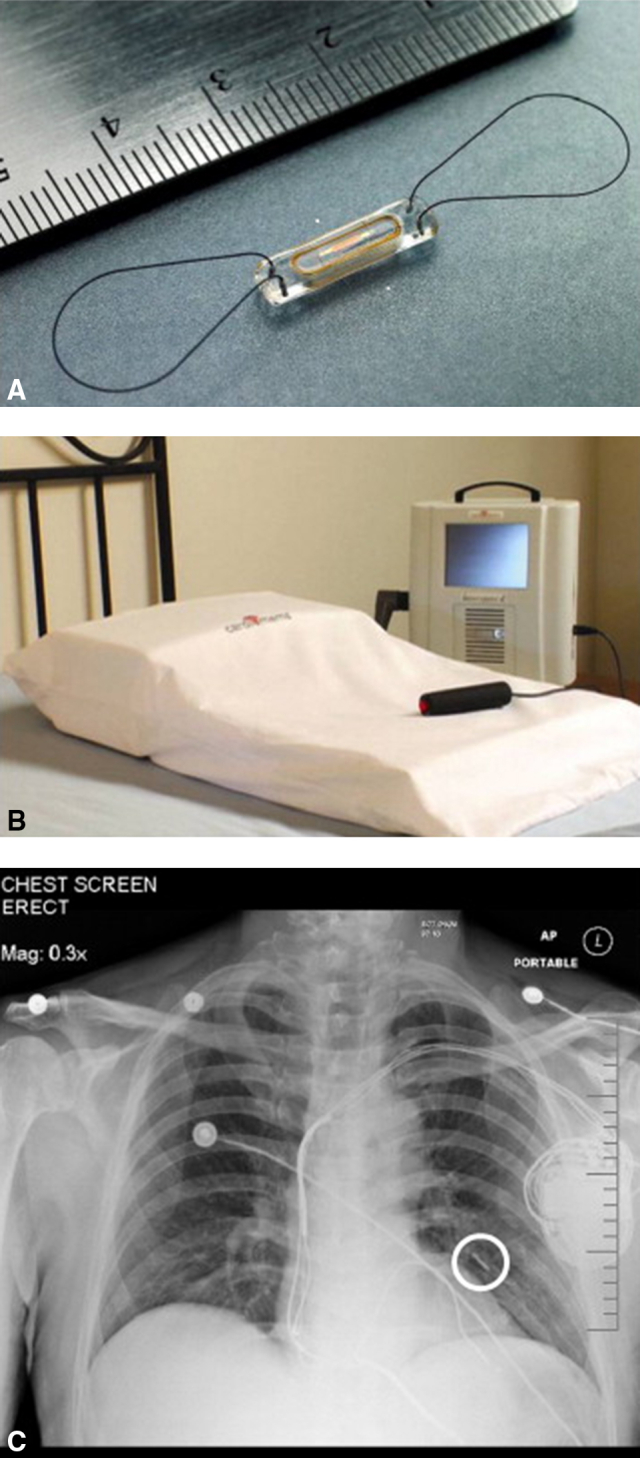
CardioMEMS (Abbott, Abbott Park, Ill) noninvasive hemodynamic monitoring system. A, Close-up view of the CardioMEMS device. B, Wireless pressure monitoring system. C, Chest radiograph of the position of the CardioMEMS device in the left pulmonary artery (*circle*).

Adapted from: Abraham WT, Adamson PB, Hasan A, Bourge RC, Pamboukian SV, Aaron MF, et al. Safety and accuracy of a wireless pulmonary artery pressure monitoring system in patients with heart failure. *Am Heart J.* 2011;161:558-66.

Utilizing remotely monitored IHM in patients with an LVAD provides several theoretical benefits both before and after implantation. Before implant, it may facilitate the earlier identification of potential LVAD patients and favorably influence preimplant hemodynamic parameters. After implantation, it provides the ability to continuously monitor patient status and holds the potential to improve post-LVAD speed optimization, identify abnormal hemodynamic status before they become symptomatic or trigger alarms, target therapeutic interventions, reduce hospitalizations and adverse events, and improve patients' functional capacity and quality of life. However, to date there are limited data on the efficacy of such a strategy to support widespread use of IHM in preoperative or postoperative LVAD patients.

Of the 550 patients with heart failure implanted with a CardioMEMS device in the CardioMEMS HF Sensor Allows Monitoring of Pressures to Improve Outcomes in NYHA Functional Class III Heart Failure Patients (CHAMPION) trial, 27 required LVAD implantation during the study follow-up period (15 in the treatment arm and 12 in the control arm).^18^ Patients in the treatment arm had a trend toward shorter time to LVAD implantation. Patients who went on to LVAD also were noted to have persistently elevated PAP despite medication changes, suggesting that CardioMEMS could assist in identifying potential nonresponders to medical therapy and trigger earlier LVAD referral. Failure of PAP to respond to aggressive adjustments in guideline-directed medical therapy can alert providers to advanced heart failure before progression to biventricular failure or frank cardiogenic shock and thus lead to earlier implantation that, in turn, may lead to improved LVAD outcomes. After LVAD implantation, PAP declined significantly all patients, but the magnitude of decline was higher in patients with noninvasive hemodynamic monitoring. A recent pilot study compared 10 consecutive patients who received CardioMEMS implant before HeartMate 3 (Abbott) LVAD with 20 historical controls.^19^ The primary outcome of a 1-year composite of all-cause mortality, acute kidney injury or need for renal replacement therapy, or RV failure occurred in 50% of the CardioMEMS group compared with 60% of the historical control group patients. In the CardioMEMS group, the primary outcome occurred in 83% of patients with elevated PAP compared with no patients with normal PAP. An example of the use of CardioMEMS in a patient with heart failure who subsequently had mechanical support and eventually cardiac transplant is seen in Figure 2. Elevated PAPs over time can be seen before the LVAD, they improve after implantation, and are maintained at an acceptable level before cardiac transplantation.

**Figure 2:**
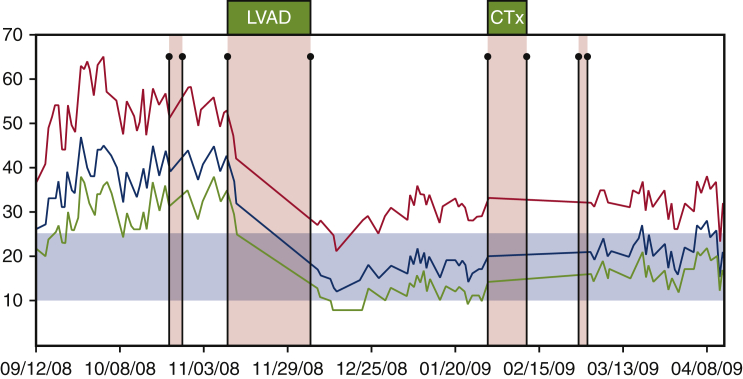
Single patient with pulmonary arterial monitor before mechanical support, during left ventricular assist device (*LVAD*) support and after cardiac transplantation (*CTx*). Systolic pulmonary artery pressure (*red*), mean pulmonary artery pressure (*blue*), and diastolic pulmonary artery pressures (*green*).

Reprinted from reference 18.

Persistently elevated PAP is undoubtedly a marker of worse disease and a poor prognostic sign in patients with chronic heart failure, and data suggest the same may be true in those receiving mechanical circulatory support. LVAD patients with normalized PAP appear to have fewer adverse events, improved quality of life, and improved survival. Although the concept of ambulatory noninvasive PAP monitoring to optimize LVAD management and improve outcomes is intriguing and may be superior to a single invasive measurement, the influence of the long-term use of IHM on outcomes or even which patients may benefit and to what degree remains uncertain. Potential disadvantages include the cost of the device, particularly if placed routinely around the time of implant, and the risks of overreacting to data resulting in an increase in patient, clinic, and coordinator burden. However, larger numbers of patients will need to be studied, ideally in a randomized trial, to determine the efficacy of IHM in the long-term management of patients with an LVAD.

Beyond the ability to follow noninvasive hemodynamic parameters to optimize pump speed and medical therapy, knowledge of hemodynamic patterns over time may give further insight into a patient's status and perhaps provide forewarning of major adverse events. Whereas pump thrombosis is typically an acute or subacute phenomenon, it may develop more subtly over time and be detected from slowly increasing LV filling pressures.^20^ Further, outflow graft obstruction or kinking, which may affect pump flow, may be suggested by increases in filling pressures in the absence of changes in pump speed, diuretics, or afterload. More commonly, increased afterload from poor blood pressure control may also manifest as elevated filling pressures.^21^ Patients may not follow blood pressure measurements at home or may have difficulty measuring them accurately; thus, knowledge of a patient's hemodynamic status over time may be helpful in focusing clinicians' efforts to manage blood pressure. Lastly, the development of aortic insufficiency can negatively influence pump function, with increasing fractions of the cardiac output regurgitating from the aortic root back into to the LV and then to the pump, creating inadequate forward flow and elevated LV filling pressures.^7^ Even with the relative frequency of transthoracic echocardiographic assessments of patients with LVADs, the quantification of aortic insufficiency can be difficult and often underestimated in the classic transthoracic views used for patients without LVADs.^22^ Increases in LV filling pressures over time may be detected by IHM and prompt a more comprehensive quantification of the degree of aortic insufficiency.

As with the invasive hemodynamic ramp, there is currently insufficient data to recommend the routine use of IHM in the LVAD population. However, it could be considered in select LVAD patients, such as those who continue to have frequent heart failure hospitalizations or frequent alarms triggered by abnormal loading conditions. In patients where an IHM was implanted as part of heart failure management before LVAD placement, continued monitoring after implant would seem reasonable. Additionally, patients implanted with an LVAD as a bridge to candidacy for transplant due to pulmonary hypertension may benefit from IHM-guided monitoring.

Although monitoring hemodynamic status noninvasively after LVAD implant is not standard care, there are intriguing opportunities to utilize such technologies to inform LVAD function, rather just monitor it (Figure 3). LVADs operate at a fixed speed and although responsive to preload and afterload, deliver a cardiac output over a relatively narrow range. For active patients, this cardiac output may be inadequate at higher workloads and impair functional capacity. A recent study of invasive hemodynamics in patients with an LVAD demonstrated substantial increases in overall cardiac output with exercise, but only a modest proportion was due to an increase in LVAD flow, the rest being attributed to contribution from the native LV. In addition, there was a concomitant increase in both left and right sided filling pressures with exercise and evidence of limited RV contractile reserve.^23^ Rather than being passively measured, future smart LVADs could incorporate real-time noninvasive hemodynamic monitoring coupled with other algorithms to assess patient activity and lead to automatic increases in pump speed to further augment cardiac output and maintain low filling pressures, more closely mimicking the native heart. Although this concept remains largely theoretical, there has been early work attempting to identify noninvasive parameters that correlate with filling pressures and could be utilized in future smart LVADs.^24^ As more patients live for longer periods of time on mechanical support,^25^ the integration of these technologies and incorporation of physiologic data into pump operation may provide better exercise tolerance and quality of life.

**Figure 3:**
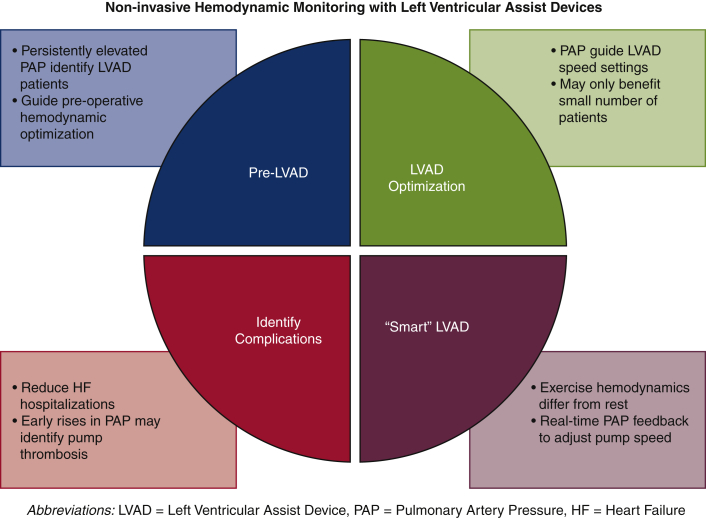
Potential uses of noninvasive hemodynamic monitoring with left ventricular assist devices (*LVADs*). *PAP*, Pulmonary artery pressure; *HF*, heart failure.

## Conclusions

Echocardiographic LVAD speed optimization, although generally effective, is often inadequate, particularly in the presence of recurrent heart failure readmissions. Invasive hemodynamic assessments have shown promise to achieve more optimal LVAD hemodynamic status, reduce readmissions, and reduce the incidence of certain major adverse events. Similarly, IHMs have been demonstrated to be useful in patients with heart failure and may also serve a role in LVAD optimization and hold the promise of improving the long-term management of patients with LVADs. However, there is currently insufficient evidence for the routine use of IHMs, but they may have use in select LVAD patients and in the future may be incorporated into the development of more physiologically responsive devices. We await data on the utility of IHM as its use increases in heart failure populations and from future clinical trials in patients with LVADs.

### Conflict of Interest Statement

Dr Teuteberg is on the advisory board for Abiomed and Takeda; is on the adverse event committee and is a speaker for Abbott; is on the advisory board and a speaker for Medtronic and CareDx; and is a speaker for Paragonix. Dr Lampert reported no conflicts of interest.

The *Journal* policy requires editors and reviewers to disclose conflicts of interest and to decline handling or reviewing manuscripts for which they may have a conflict of interest. The editors and reviewers of this article have no conflicts of interest.

## References

[1] Aaronson K.D., Slaughter M.S., Miller L.W., McGee E.C., Cotts W.G., Acker M.A. (2012). Use of an intrapericardial, continuous-flow, centrifugal pump in patients awaiting heart transplantation. Circulation.

[2] Mehra M.R., Uriel N., Naka Y., Cleveland J.C., Yuzefpolskaya M., Salerno C.T. (2019). A fully magnetically levitated left ventricular assist device - final report. N Engl J Med.

[3] Slaughter M.S., Rogers J.G., Milano C.A., Russell S.D., Conte J.V., Feldman D. (2009). Advanced heart failure treated with continuous-flow left ventricular assist device. N Engl J Med.

[4] Molina E.J., Shah P., Kiernan M.S., Cornwell W.K., Copeland H., Takeda K. (2021). The Society of Thoracic Surgeons intermacs 2020 annual report. Ann Thorac Surg.

[5] Feldman D., Pamboukian S.V., Teuteberg J.J., Birks E., Lietz K., Moore S.A. (2013). The 2013 International Society for Heart and Lung Transplantation guidelines for mechanical circulatory support: executive summary. J Heart Lung Transplant.

[6] Teuteberg J.J., Slaughter M.S., Rogers J.G., McGee E.C., Pagani F.D., Gordon R. (2015). The HVAD left ventricular assist device: risk factors for neurological events and risk mitigation strategies. JACC Heart Fail.

[7] Cowger J., Rao V., Massey T., Sun B., May-Newman K., Jorde U. (2015). Comprehensive review and suggested strategies for the detection and management of aortic insufficiency in patients with a continuous-flow left ventricular assist device. J Heart Lung Transplant.

[8] Pinney S.P., Anyanwu A.C., Lala A., Teuteberg J.J., Uriel N., Mehra M.R. (2017). Left ventricular assist devices for lifelong support. J Am Coll Cardiol.

[9] Mudd J.O., Cuda J.D., Halushka M., Soderlund K.A., Conte J.V., Russell S.D. (2008). Fusion of aortic valve commissures in patients supported by a continuous axial flow left ventricular assist device. J Heart Lung Transplant.

[10] Moazami N., Fukamachi K., Kobayashi M., Smedira N.G., Hoercher K.J., Massiello A. (2013). Axial and centrifugal continuous-flow rotary pumps: a translation from pump mechanics to clinical practice. J Heart Lung Transplant.

[11] Uriel N., Levin A.P., Sayer G.T., Mody K.P., Thomas S.S., Adatya S. (2015). Left ventricular decompression during speed optimization ramps in patients supported by continuous-flow left ventricular assist devices: device-specific performance characteristics and impact on diagnostic algorithms. J Card Fail.

[12] Uriel N., Sayer G., Addetia K., Fedson S., Kim G.H., Rodgers D. (2016). Hemodynamic ramp tests in patients with left ventricular assist devices. JACC Heart Fail.

[13] Imamura T., Jeevanandam V., Kim G., Raikhelkar J., Sarswat N., Kalantari S. (2019). Optimal hemodynamics during left ventricular assist device support are associated with reduced readmission rates. Circ Heart Fail.

[14] Uriel N., Burkhoff D., Rich J.D., Drakos S.G., Teuteberg J.J., Imamura T. (2019). Impact of hemodynamic ramp test-guided HVAD speed and medication adjustments on clinical outcomes. Circ Heart Fail.

[15] Abraham W.T., Adamson P.B., Bourge R.C., Aaron M.F., Costanzo M.R., Stevenson L.W. (2011). Wireless pulmonary artery haemodynamic monitoring in chronic heart failure: a randomised controlled trial. Lancet.

[16] Lampert B.C., Emani S. (2015). Remote hemodynamic monitoring for ambulatory left ventricular assist device patients. J Thorac Dis.

[17] Veenis J.F., Brugts J.J. (2020). Remote monitoring for better management of LVAD patients: the potential benefits of CardioMEMS. Gen Thorac Cardiovasc Surg.

[18] Feldman D.S., Moazami N., Adamson P.B., Vierecke J., Raval N., Shreenivas S. (2018). The utility of a wireless implantable hemodynamic monitoring system in patients requiring mechanical circulatory support. ASAIO J.

[19] Veenis J.F., Radhoe S.P., van Mieghem N.M., Manintveld O.C., Bekkers J.A., Caliskan K. (2021). Safety and feasibility of hemodynamic pulmonary artery pressure monitoring using the CardioMEMS device in LVAD management. J Card Surg.

[20] Najjar S.S., Slaughter M.S., Pagani F.D., Starling R.C., McGee E.C., Eckman P. (2014). An analysis of pump thrombus events in patients in the HeartWare ADVANCE bridge to transplant and continued access protocol trial. J Heart Lung Transplant.

[21] Lampert B.C., Eckert C., Weaver S., Scanlon A., Lockard K., Allen C. (2014). Blood pressure control in continuous flow left ventricular assist devices: efficacy and impact on adverse events. Ann Thorac Surg.

[22] Grinstein J., Kruse E., Sayer G., Fedson S., Kim G.H., Sarswat N. (2016). Novel echocardiographic parameters of aortic insufficiency in continuous-flow left ventricular assist devices and clinical outcome. J Heart Lung Transplant.

[23] Tran T., Muralidhar A., Hunter K., Buchanan C., Coe G., Hieda M. (2021). Right ventricular function and cardiopulmonary performance among patients with heart failure supported by durable mechanical circulatory support devices. J Heart Lung Transplant.

[24] Grinstein J., Rodgers D., Kalantari S., Sayer G., Kim G.H., Sarswat N. (2018). HVAD waveform analysis as a noninvasive marker of pulmonary capillary wedge pressure: a first step toward the development of a smart left ventricular assist device pump. ASAIO J.

[25] Teuteberg J.J., Cleveland J.C., Cowger J., Higgins R.S., Goldstein D.J., Keebler M. (2020). The Society of Thoracic Surgeons intermacs 2019 annual report: the changing landscape of devices and indications. Ann Thorac Surg.

